# Tension Pneumoperitoneum Attributable to COVID-19

**DOI:** 10.4269/ajtmh.21-0254

**Published:** 2021-06-17

**Authors:** Hiroki Matsuura, Atsushi Okita

**Affiliations:** 1 Department of General Internal Medicine, Okayama City Hospital, Okayama, Japan;; 2 Department of Surgery, Okayama City Hospital, Okayama, Japan

**Figure 1.**
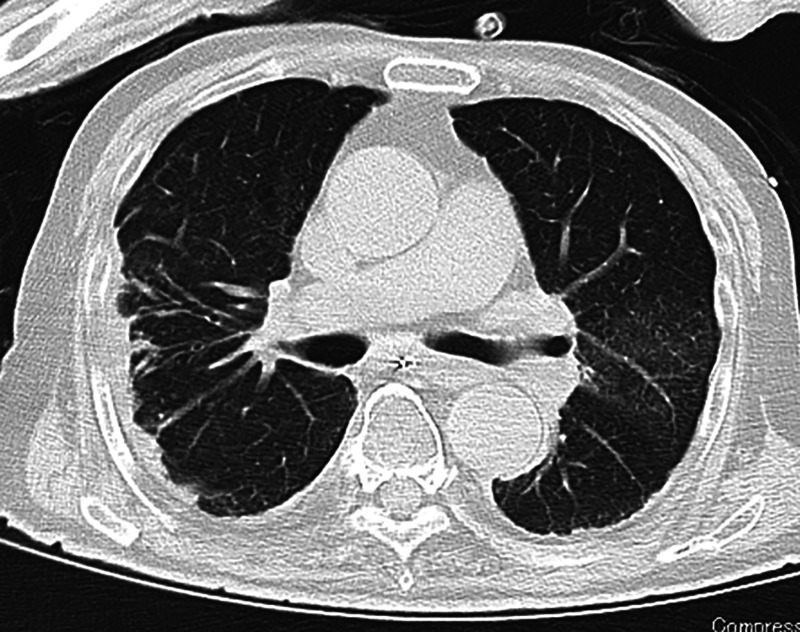
Chest computed tomography (CT) demonstrates multifocal and peripheral ground-glass opacities with predominant consolidation in the mid to lower lung.

**Figure 2.**
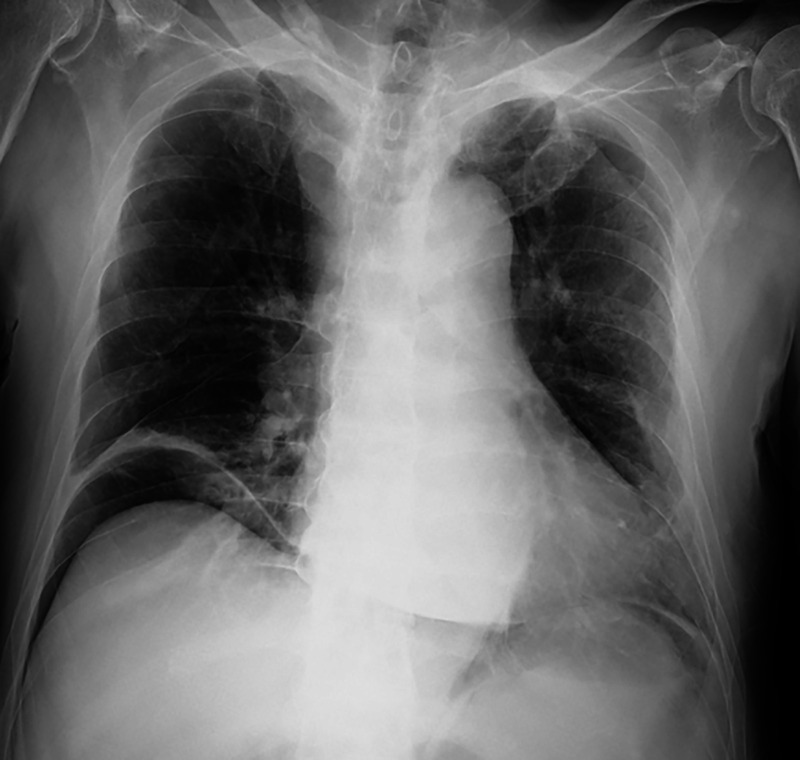
Chest radiograph shows free gas in the bilateral subdiaphragmatic region.

**Figure 3.**
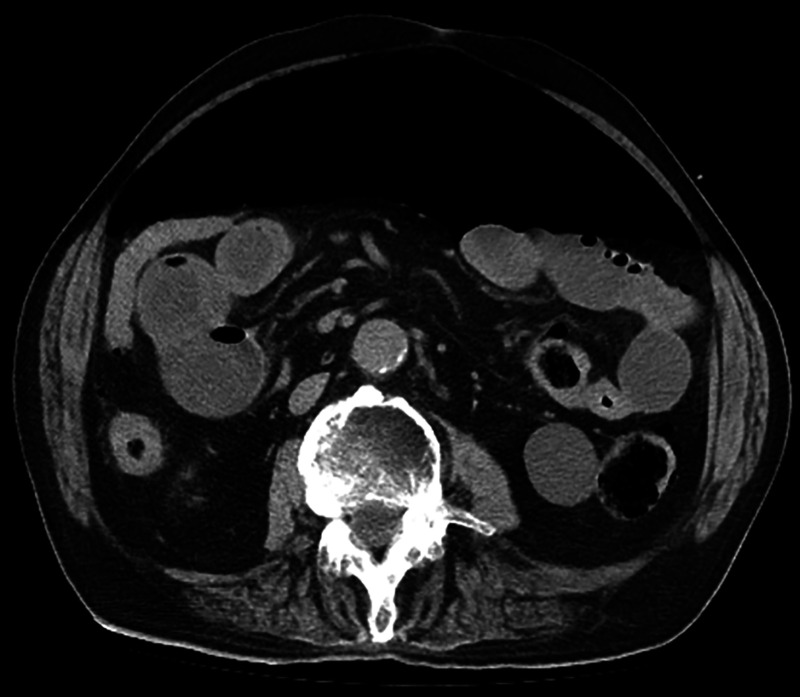
Enhanced computed tomography (CT) reveals a large amount of free air in the abdomen without any signs of gastrointestinal perforation.

An 84-year-old man was admitted to our hospital with coronavirus disease 2019 (COVID-19). His medical history included mild hypertension and diabetes mellitus. He was febrile, but his other vital signs were stable. On admission, computed tomography (CT) revealed multifocal and peripheral ground-glass opacities with predominant consolidation in the mid to lower lung (Figure 1). Three days after admission, he reported sudden severe dyspnea, nausea, and abdominal distension. His temperature was 37.4°C, blood pressure was 98/64 mmHg, respiratory rate was 35 breaths/minute, pulse was 118/minute, and oxygen saturation was 92% with 4-liter nasal prongs (84% with room air). Physical findings revealed progressive abdominal discomfort without pain, guarding, or rebound tenderness. A chest radiograph showed free subdiaphragmatic air bilaterally (Figure 2). Repeat enhanced CT demonstrated a large amount of free air in the abdomen without intra-abdominal fluid collection, pneumothorax, or pneumomediastinum (Figure 3). Emergency percutaneous needle decompression with an 18-G venous catheter was performed. His dyspnea improved immediately without remission after the procedure. Subsequently, laboratory examinations showed no significant changes suggestive of intestinal perforation. Based on the clinical and laboratory findings, we diagnosed COVID-19 with tension pneumoperitoneum. Because there were no abdominal symptoms or radiologic findings of visceral perforation, conservative management was continued. His respiratory symptoms improved gradually. He was discharged after 7 days without remission. Tension pneumoperitoneum is a life-threatening form of pneumoperitoneum requiring urgent surgical intervention. The pathophysiology of tension pneumoperitoneum is associated with an abrupt increase in intra-abdominal pressure caused by infusion of a large amount gas. Increased intra-abdominal pressure elevates the diaphragm, reduces lung volume, and causes ventilator insufficiency. It also leads to decreased cardiac output with decreasing venous return and diastolic filling. Most reported cases of tension pneumoperitoneum involve complications during upper or lower gastrointestinal endoscopy, improper intubation, and cardiopulmonary resuscitation.^1^^,^^2^ However, the patient had no clinical history suggesting the infusion of a large amount of gas into the gastrointestinal tract or peritoneal cavity. The mechanism of pneumoperitoneum attributable to COVID-19 remains unknown. Similar to other respiratory infections related to alveolar injury, alveolar rupture attributable to COVID-19 infection causes cystic formations within the lungs. It is postulated that increased respiratory effort and persistent cough generate severe intrapulmonary pressure, which lead to the rupture of alveolar cysts. The rupture of cysts causes air to escape from pulmonary tissue. Occasionally, this escaped air can dissect along the perivascular and peribronchial vascular sheath into the retroperitoneum, leading to pneumoperitoneum. Severe cases of COVID-19 can progress to acute respiratory distress syndrome, disseminated intravascular coagulation, venous thromboembolism, acute myocarditis, acute kidney failure, Kawasaki disease, Guillain-Barre syndrome, and multi-organ failure.^3^ Pneumoperitoneum is a relatively rare complication of COVID-19 compared with pulmonary complications.^4^ It is unknown whether spontaneous pneumoperitoneum of COVID-19 occurs without active air infusion. We performed needle decompression using abdominal CT imaging. However, CT evaluation during an emergent condition is not mandatory because it can lead to delayed surgical intervention. Tension pneumoperitoneum has progressive, irreversible consequences and results in death. Patients with severe COVID-19 have reduced respiratory functional reserves, and their condition can deteriorate rapidly. Early detection and appropriate management of tension pneumoperitoneum are important for preventing serious complications.
